# Machine learning techniques in disease forecasting: a case study on rice blast prediction

**DOI:** 10.1186/1471-2105-7-485

**Published:** 2006-11-03

**Authors:** Rakesh Kaundal, Amar S Kapoor, Gajendra PS Raghava

**Affiliations:** 1 Bioinformatics Centre, Institute of Microbial Technology, Sector 39-A, Chandigarh 160036, India.; 2 Department of Plant Pathology, CSK HPAU, Palampur HP 176062, India.

## Abstract

**Background:**

Diverse modeling approaches *viz*. neural networks and multiple regression have been followed to date for disease prediction in plant populations. However, due to their inability to predict value of unknown data points and longer training times, there is need for exploiting new prediction softwares for better understanding of plant-pathogen-environment relationships. Further, there is no online tool available which can help the plant researchers or farmers in timely application of control measures. This paper introduces a new prediction approach based on support vector machines for developing weather-based prediction models of plant diseases.

**Results:**

Six significant weather variables were selected as predictor variables. Two series of models (cross-location and cross-year) were developed and validated using a five-fold cross validation procedure. For cross-year models, the conventional multiple regression (REG) approach achieved an average correlation coefficient (*r*) of 0.50, which increased to 0.60 and percent mean absolute error (%MAE) decreased from 65.42 to 52.24 when back-propagation neural network (BPNN) was used. With generalized regression neural network (GRNN), the *r *increased to 0.70 and %MAE also improved to 46.30, which further increased to *r *= 0.77 and %MAE = 36.66 when support vector machine (SVM) based method was used. Similarly, cross-location validation achieved *r *= 0.48, 0.56 and 0.66 using REG, BPNN and GRNN respectively, with their corresponding %MAE as 77.54, 66.11 and 58.26. The SVM-based method outperformed all the three approaches by further increasing *r *to 0.74 with improvement in %MAE to 44.12. Overall, this SVM-based prediction approach will open new vistas in the area of forecasting plant diseases of various crops.

**Conclusion:**

Our case study demonstrated that SVM is better than existing machine learning techniques and conventional REG approaches in forecasting plant diseases. In this direction, we have also developed a SVM-based web server for rice blast prediction, a first of its kind worldwide, which can help the plant science community and farmers in their decision making process. The server is freely available at .

## Background

Weather-based forecasting systems reduce the cost of production by optimizing the timing and frequency of application of control measures and ensures operator, consumer and environmental safety by reducing chemical usage. A major aim of many forecasting systems is to reduce fungicide use, and accurate prediction is important to synchronize the use of disease control measures to avoid crop losses [1]. A prediction model based on the relationship between environmental conditions at the time of management and late-season disease severity could be used to guide management decisions. Thus, if a sound forewarning system is developed, the explosive nature of the disease could be prevented by timely application of the control measures. Various techniques of computer modeling and simulation *viz*. machine learning techniques like artificial neural networks and the conventional multiple regression approaches are being used to help synthesize and develop scientists' understanding of this complex plant-pathogen-environment relationship. The resultant models enable exploration of the factors that govern disease epidemics and the design of control systems that minimize yield losses. The same models have potential to guide breeding programs and work to develop strategies that will prolong the usefulness of disease-resistance genes. Thus, we undertook the present case study on rice blast disease forecasting by following a new prediction approach, support vector machine and compared its performance with the existing artificial neural networks-based and multiple regression-based prediction approaches.

Rice (*Oryza sativa *L.) is the single most important food crop for more than one-third of the world's population. Of the various diseases limiting rice productivity, blast disease caused by *Pyricularia grisea *Sacc. {*Magnaporthe grisea *(Hebert) Barr.} continues to be an enigmatic problem in several rice growing ecosystems of both tropical and temperate regions of the world and is a serious constraint in realizing the full yield potential of rice cultivars. It continues to be the most destructive disease of rice despite decades of research towards its control. Weather has a very important role to play in the appearance, multiplication and spread of the blast fungus. Considerable efforts have been directed towards developing blast-resistant cultivars, but due to high variability in blast pathogen, most of the resistant varieties frequently succumb to this disease. Therefore, the most practical way to control blast epidemics have been the use of fungicides. However, due to high cost of chemicals as well as their hazardous effects, use of fungicides is invariably uneconomic. Moreover, farmers are sometimes forced to skip the actual date of fungicide application due to lack of knowledge regarding the actual time of appearance of the disease. Therefore, for the judicious use of fungicides, forewarning of blast is very important.

Previous attempts to describe the relationship between rice blast severity and environmental conditions have been made in various countries through both the empirical and explanatory simulation models developed only through the conventional regression analysis *viz*. in Japan [2,3], Korea [4-6], China [7], Taiwan [8], India [9-11], Thailand [12] and the Philippines [13,14]. However, very limited use of these models have been implemented by farmers to manage rice blast because of two plausible reasons: firstly, growers/farmers tend to be risk-aversed and are not properly convinced on the use of disease forecasting tools, and secondly, the mathematical relationships between the environmental conditions and the specific stages of rice blast infection cycle are not fully understood. This makes conventional modeling approaches such as multiple regression difficult.

Various other attempts to establish a quantitative relationship between weather and disease infection from field studies have not always been successful. Davis *et al*. did not find any obvious relationship between disease severity and weather, and infection could not be associated with any specific sequence of weather events [15]. Using multiple linear regression (REG) analysis, Chakraborty and Billard showed that REG model was not equally effective in predicting infection events in the 2 years of their field study in Australia [16]. Subsequently, another independent study has shown that the REG model does not adequately explain infection at a field site in Queensland, Australia [17]. These studies indicate that, although the REG models were able to explain disease development at a particular field site for certain years, its ability to generalize across field sites and years was poor. Diverse modeling approaches have therefore, been followed for advanced understanding (i.e. predictability) of phenomena associated with disease in plant populations. Till date, artificial neural networks (ANNs) have been reported to be the good alternate to the conventional multiple regression techniques as ANNs are reputed to excel at extracting sometimes subtle patterns from large multivariate data sets without preconceived assumptions about model form due to incompletely understood, possibly complex, relationships [18-23]. The next major advancement was a feedback function of ANN that adjusted weights to minimal error values; however, popularization of ANNs as a distinct class of models occurred in the 1980s when the activation threshold was replaced by a continuous function and a multilayer network took derivatives from a backpropagation of errors to approximate the target output by nonlinear functions [24]. Another alternative to back-propagation that has been widely used in regression problems is the generalized regression neural network, which involves one-pass learning algorithm with a highly parallel structure that provides estimates of continuous variables and converges to the underlying (linear or nonlinear) regression surface [25]. Both these backpropagation neural network (BPNN) and generalized regression neural network (GRNN) architectures has remained a mainstay among the models in the ANN family.

However, both these ANN architectures have there own limitations. One disadvantage of backpropagation is that it can take a large number of iterations to converge to the desired solution. It also requires very long training time and is subject to converging to local minima instead of finding the global minimum error surface. For a mature application, the long training time may be justified. To guard against getting trapped in local minima, the BPNN can be compared with the GRNN accuracy as it is fast learning and have ability to convergence to the optimal regression surface as the number of samples becomes very large [25]. However, GRNN also requires substantial computation to evaluate new points. It trains almost instantly, but tends to be large and slow. GRNN also does not extrapolate i.e. its inability in predicting the value of unknown data points by projecting a function beyond the range of known data points [25-27]. Thus, there is an urgent need of exploiting the latest upcoming prediction softwares for better and improved understanding of the plant-pathogen-environment relationships.

This paper introduces a new prediction method based on a powerful machine learning technique Support Vector Machines (SVM) originally developed by Vapnik and co-workers at Bell Laboratories as a very effective method for general-purpose supervised predictions [28-30]. It has been shown in the past that this machine learning technique is very effective in the classification of proteins particularly in discriminating membrane proteins [31], prediction of subcellular localization [32-34], solvent accessibility [35], CTL epitopes [36], binding peptides [37], protein structures [38], protein-protein binding sites [39] and gene expression level [40]. SVM provides an alternative or complement to the present ANN and REG based approaches for model development. The SVM learns how to classify from a training set of feature vectors, whose expected outputs are already known. The training enables a binary classifying SVM to define a plane in the feature space, which optimally separates the training vectors of two classes. When a new feature vector is fed, its class is predicted on the basis of which side of the plane it maps. To the best of authors' knowledge, there is no report of using support vector machines in understanding the relationship between disease severity and its associated environmental conditions.

Hence, the present case study was aimed to determine the usefulness of SVM models over the existing artificial neural network and conventional multiple regression models to predict rice blast severity based on prevailing weather conditions both within and between the locations/years, and to calculate the overall risk of rice blast infection at these field sites using a set of weights from the trained SVM models. For this, we used 5-year rice blast/weather data collected from five different locations spread over the district Kangra of Himachal Pradesh (India) as part of a National Agricultural Technology Project (NATP) which was implemented at CSK Himachal Pradesh Agricultural University, Palampur, Himachal Pradesh (India). This paper reports the first ever disease prediction models as well as web-based server developed using SVM, worldwide.

## Results

### Cross-location model development and validation

Within each year, cross-location models were developed and validated separately with REG, BPNN, GRNN and SVM approaches (Table 1). In the year 2000, when cross-location multiple regression models were validated against each other, the coefficient of determination (r^2^) value went maximum up to 0.40 with its corresponding percent mean absolute error (% MAE) value of 62.92 (location-II as test data); however, with BPNN-based validation, the r^2 ^improved by about 21% to 0.61 with almost 36% improvement in its corresponding % MAE value (26.60); which further increased to r^2 ^= 0.79 with improvement in its respective % MAE (25.81), when GRNN-based validation approach was applied. When SVM-based validation approach was performed, the r^2 ^improved by about 9% to 0.88, with also 9% decrease in its % MAE value (17.29). In 2001, the maximum r^2 ^value observed was 0.61 with 59.77% mean absolute error with REG based validation (location-IV as test data), whereas the r^2 ^improved to 0.62 with improvement in its respective % MAE value also (57.34) with BPNN approach which further increased by 17% to r^2 ^= 0.79 with 24% improvement in its corresponding % MAE (33.11) when GRNN-based validation approach was followed. With SVM, about 11% increase in coefficient of determination (r^2 ^= 0.90) corresponded to about 9% improvement in percent mean absolute error (%MAE = 24.16). In 2002, with REG-based validation, maximum r^2 ^reached up to 0.50 with its respective % MAE value as 110.72 (location-IV as test data), whereas the r^2 ^value went maximum up to 0.74 with drastic improvement in its respective % MAE value (84.29) when validated with BPNN-based approach (location-V as test data) which further improved to r^2 ^= 0.87 and % MAE = 82.64 with GRNN-based validation approach. The r^2 ^increased drastically to 0.98 with improvement in its corresponding % MAE to 51.54 when SVM-based validation approach was performed (location-IV as test data). In 2003, maximum r^2 ^reached up to 0.48 having corresponding % MAE value of 69.78 with REG based validation (location-II as test data), however with BPNN-based validation, the r^2 ^improved to 0.49 with improvement in its % MAE (61.70) also. Maximum r^2 ^and % MAE achieved with GRNN was 0.76 and 49.57 which was respectively about 27% and 12% better than BPNN (location-III as test data). With SVM, the r^2 ^slightly increased to 0.77 with drastic improvement of about 30% in its corresponding % MAE to 19.76. Similarly in 2004, the maximum r^2 ^value attained with REG based validation was 0.46 with its corresponding % MAE value of 78.63 (location-IV as test data), whereas with BPNN-based validation, an 8% increase in r^2 ^(0.52) corresponded to about 33% improvement in its respective % MAE value (45.20) which with GRNN, again increased to r^2 ^= 0.58 with decrease in its corresponding % MAE (37.10). As observed earlier, the SVM again outperformed all the three other approaches with about 16% increase in r^2 ^to 0.74 as well as improvement in its corresponding mean absolute error to 31.67%.

**Table 1:** Comparison of multiple regression (REG), backpropagation neural network (BPNN), generalized regression neural network (GRNN) and support vector machine (SVM) based prediction accuracy of rice blast severity measured as correlation coefficient (r), coefficient of determination (r^2^) and percent mean absolute error (%MAE) of the observed value for 'cross-location' models over various years.

**Year(s)**	**Location(s)**		**Multiple Regression (REG)**			**Artificial Neural Network (ANN)**						**Support Vector Machine (SVM)**		
						**BPNN**			**GRNN**					

	**Training Data**	**Test Data**	**r**	**r**^**2**^	**%MAE**	**r**	**r**^**2**^	**%MAE**	**r**	**r**^**2**^	**%MAE**	**r**	**r**^**2**^	**%MAE**

2000	L-I, L-II, L-III, L-IV	L-V	0.62	0.38	75.12	0.51	0.26	51.94	0.62	0.38	37.83	0.62	0.38	37.42
	L-I, L-II, L-III, L-V	L-IV	0.59	0.35	99.77	0.69	0.48	72.75	0.60	0.36	41.39	0.69	0.48	39.41
	L-I, L-II, L-V, L-IV	L-III	0.57	0.33	39.20	0.60	0.36	49.47	0.75	0.56	40.35	0.84	0.71	23.71
	L-I, L-V, L-III, L-IV	L-II	0.63	0.40	62.92	0.78	0.61	26.60	0.89	0.79	25.81	0.94	0.88	17.29
	L-V, L-II, L-III, L-IV	L-I	0.44	0.19	58.01	0.48	0.23	57.62	0.51	0.26	55.62	0.54	0.29	50.88
														
2001	L-I, L-II, L-III, L-IV	L-V	0.39	0.15	89.27	0.66	0.44	59.17	0.95	0.90	95.08	0.98	0.96	66.40
	L-I, L-II, L-III, L-V	L-IV	0.78	0.61	59.77	0.79	0.62	57.34	0.89	0.79	33.11	0.95	0.90	24.16
	L-I, L-II, L-V, L-IV	L-III	-0.24	0.06	73.18	0.21	0.04	59.45	0.41	0.17	50.50	0.51	0.26	35.30
	L-I, L-V, L-III, L-IV	L-II	0.50	0.25	52.35	0.45	0.20	59.50	0.52	0.27	48.87	0.63	0.40	42.56
	L-V, L-II, L-III, L-IV	L-I	-0.27	0.07	115.20	0.38	0.14	96.66	0.34	0.12	94.98	0.41	0.17	76.22
														
2002	L-I, L-II, L-IV	L-V	0.51	0.26	94.75	0.86	0.74	84.29	0.93	0.87	82.64	0.82	0.67	36.09
	L-I, L-II, L-V	L-IV	-0.71	0.50	110.72	0.72	0.52	79.11	0.72	0.52	57.87	0.99	0.98	51.54
	L-I, L-V, L-IV	L-II	0.61	0.37	76.15	0.64	0.41	69.48	0.69	0.48	67.63	0.81	0.66	45.36
	L-V, L-II, L-IV	L-I	-0.15	0.02	108.31	-0.34	0.12	143.29	-0.30	0.09	127.32	0.12	0.01	95.39
														
2003	L-I, L-II, L-III, L-IV	L-V	0.46	0.21	67.92	0.50	0.25	53.61	0.63	0.40	49.30	0.86	0.74	42.93
	L-I, L-II, L-III, L-V	L-IV	0.14	0.02	74.96	0.46	0.21	65.17	0.51	0.26	59.29	0.86	0.74	41.80
	L-I, L-II, L-V, L-IV	L-III	0.53	0.28	68.09	0.59	0.35	58.59	0.87	0.76	49.57	0.88	0.77	19.76
	L-I, L-V, L-III, L-IV	L-II	0.69	0.48	69.78	0.70	0.49	61.70	0.72	0.52	58.39	0.78	0.61	53.17
	L-V, L-II, L-III, L-IV	L-I	0.57	0.33	53.05	0.65	0.42	51.09	0.69	0.48	49.85	0.70	0.49	49.18
														
2004	L-I, L-II, L-IV	L-V	0.18	0.03	90.55	0.63	0.40	61.81	0.73	0.53	41.01	0.83	0.69	38.86
	L-I, L-II, L-V	L-IV	0.68	0.46	78.63	0.72	0.52	45.20	0.76	0.58	37.10	0.86	0.74	31.67
	L-I, L-V, L-IV	L-II	0.39	0.15	89.98	0.10	0.01	65.18	0.44	0.19	51.82	0.69	0.48	46.54
	L-V, L-II, L-IV	L-I	0.47	0.22	54.88	0.43	0.19	69.34	0.67	0.45	67.79	0.74	0.55	40.70

where,

L – I = Location-I *viz*. Palampur (1^st ^date of transplanting; 15 days prior to normal transplanting)

L – II = Location-II *viz*. Palampur (2^nd ^date of transplanting; normal time of transplanting)

L – III = Location-III *viz*. Palampur (3^rd ^date of transplanting; 15 days after the normal transplanting)

L – IV = Location-IV *viz*. Rice Research Station, Malan (CSK HPAU)

L – V = Location-V *viz*. Farmers' fields, Pharer.

### Cross-year model development and validation

Within each location, cross-year models were also developed and validated. Here also, comparison between all the four approaches followed was made on the basis of r^2 ^and % MAE values (Table 2). All the locations showed improvement in r^2 ^values and decrease in percent mean absolute error values when models developed with SVM were validated against the test data as compared to the REG, BPNN and GRNN-based validation approaches. At location-I (Palampur-1^st ^date of transplanting), when within year cross validation was performed with REG, the r^2 ^went maximum up to 0.48 with the corresponding % MAE value of 61.68 (year 2001 as test data), whereas with BPNN approach, the r^2 ^value improved to 0.50 and the % MAE value improved to 59.73 which further increased to r^2 ^= 0.52 with its respective % MAE = 59.17 when GRNN was performed. The SVM-based validation approach showed about 9% increase in r^2 ^value (0.61) with about 10% simultaneous improvement in its % MAE (49.06). At location-II (Palampur-II^nd ^date of transplanting), maximum r^2 ^observed was 0.38 with its respective % MAE as 92.79 (2004 as test data) with REG approach, whereas the BPNN-based validation achieved about 23% higher coefficient of determination (r^2 ^= 0.61) with about 48% simultaneous improvement in its corresponding % MAE also (2000 as test data). With GRNN, the r^2 ^showed drastic increase of about 26% (0.87) with also improvement in its % MAE to 40.15, however, when SVM was followed, the r^2 ^remained constant but the % MAE improved by about 15% to 25.41 thereby again indicating the supremacy of SVM over the other three approaches. At location-III (Palampur-III^rd ^date of transplanting), with REG, maximum r^2 ^was observed to be 0.38 (2000 as test data) with a mean absolute error of 43.67%; however, the r^2 ^increased to 0.46 with also improvement in % MAE (36.30) with BPNN-based validation (2003 as test data). The GRNN again improved the r^2 ^and % MAE to 0.69 and 33.38, respectively (2000 as test data). With SVM, the r^2 ^increased by about 5% to 0.74 with also about 11% improvement in its respective % MAE (22.18). At location-IV (Rice Research Station, Malan), maximum r^2 ^value went up to 0.79 with the % MAE value of 68.55 (2001 as test data) with REG approach. With BPNN, the r^2 ^remained constant but the % MAE showed about 3% improvement (65.23). With GRNN-based validation, the r^2 ^increased slightly by about 2% to 0.81, but the % MAE improved drastically by about 35% (30.75). The r^2 ^again increased to 0.94 with improvement in its corresponding % MAE to 29.90 (2001 as test data). Similarly, at location-V (farmers' fields, Pharer), the maximum r^2 ^value observed with REG was 0.61 with the corresponding % MAE value as 72.13 (2001 as test data), whereas the r^2 ^improved by about 13% to 0.74 with its corresponding % MAE as 51.91, an error improvement of about 20% (2002 as test data) when validated with BPNN-based approach. The GRNN again proved better over REG and BPNN as the r^2 ^increased by about 7% to 0.81 with improvement in its respective % MAE value to 50.56 (2001 as test data). However, SVM again proved its supremacy over all the approaches followed by achieving drastic increase of about 15% (2000 as test data) in coefficient of determination (r^2 ^= 0.96) as well as simultaneous improvement of about 35% in its respective percent mean absolute error value (15.23).

**Table 2:** Comparison of multiple regression (REG), backpropagation neural network (BPNN), generalized regression neural network (GRNN) and support vector machine (SVM) based prediction accuracy of rice blast severity measured as correlation coefficient (r), coefficient of determination (r^2^) and percent mean absolute error (%MAE) of observed value for 'cross-year' models over various locations.

**Location(s)**	**Year (s)**		**Multiple Regression (REG)**			**Artificial Neural Network (ANN)**						**Support Vector Machine (SVM)**		
						**BPNN**			**GRNN**					

	**Training Data**	**Test Data**	**r**	**r**^**2**^	**%MAE**	**r**	**r**^**2**^	**%MAE**	**r**	**r**^**2**^	**%MAE**	**r**	**r**^**2**^	**%MAE**

L – I	2000,01,02,03	2004	0.56	0.31	56.85	0.59	0.35	56.19	0.66	0.44	50.72	0.67	0.45	46.23
	2001,02,03,04	2000	0.36	0.13	60.37	0.50	0.25	58.90	0.63	0.40	40.86	0.75	0.56	38.07
	2000,02,03,04	2001	0.69	0.48	61.68	0.71	0.50	59.73	0.72	0.52	59.17	0.78	0.61	49.06
	2000,01,03,04	2002	0.17	0.03	85.70	0.17	0.03	77.18	0.35	0.12	75.97	0.54	0.29	67.35
	2000,01,02,04	2003	0.30	0.09	65.19	0.58	0.34	53.59	0.62	0.38	52.92	0.70	0.49	39.14
														
L – II	2000,01,02,03	2004	0.62	0.38	92.79	0.11	0.01	72.18	0.49	0.24	70.58	0.66	0.44	43.52
	2001,02,03,04	2000	0.58	0.34	45.04	0.78	0.61	44.29	0.93	0.87	40.15	0.93	0.87	25.41
	2000,02,03,04	2001	0.23	0.05	77.56	0.42	0.18	73.12	0.53	0.28	62.49	0.53	0.28	50.32
	2000,01,03,04	2002	0.48	0.23	51.76	0.56	0.32	48.09	0.76	0.58	41.03	0.79	0.62	40.13
	2000,01,02,04	2003	-0.10	0.01	99.31	0.20	0.04	60.62	0.22	0.05	56.81	0.60	0.36	45.54
														
L-III	2000,01	2003	0.50	0.25	52.72	0.68	0.46	36.30	0.82	0.67	29.97	0.84	0.71	20.27
	2001,03	2000	0.62	0.38	43.67	0.60	0.36	40.62	0.83	0.69	33.38	0.86	0.74	22.18
	2000,03	2001	0.14	0.02	71.93	0.58	0.34	38.30	0.63	0.40	35.93	0.65	0.42	35.10
														
L-IV	2000,01,02,03	2004	0.66	0.44	56.99	0.62	0.38	51.72	0.78	0.61	49.62	0.84	0.71	47.49
	2001,02,03,04	2000	0.55	0.30	53.03	0.68	0.46	45.99	0.72	0.52	43.31	0.77	0.59	41.90
	2000,02,03,04	2001	0.89	0.79	68.55	0.89	0.79	65.23	0.90	0.81	30.75	0.97	0.94	29.90
	2000,01,03,04	2002	0.84	0.71	91.18	0.90	0.81	23.93	0.94	0.88	20.95	0.96	0.92	14.79
	2000,01,02,04	2003	0.48	0.23	71.71	0.56	0.31	67.39	0.58	0.34	62.60	0.66	0.44	40.39
														
L-V	2000,01,02,03	2004	0.38	0.14	78.13	0.64	0.41	62.55	0.66	0.44	56.82	0.80	0.64	50.22
	2001,02,03,04	2000	0.67	0.45	55.71	0.71	0.51	38.99	0.73	0.53	32.09	0.98	0.96	15.23
	2000,02,03,04	2001	0.78	0.61	72.13	0.83	0.69	52.07	0.90	0.81	50.56	0.93	0.87	21.18
	2000,01,03,04	2002	0.53	0.28	56.44	0.86	0.74	51.91	0.87	0.76	46.30	0.87	0.76	35.28
	2000,01,02,04	2003	0.61	0.37	54.91	0.61	0.37	50.26	0.62	0.38	48.17	0.65	0.42	46.17

where,

L – I = Location-I *viz*. Palampur (1^st ^date of transplanting; 15 days prior to normal transplanting)

L – II = Location-II *viz*. Palampur (2^nd ^date of transplanting; normal time of transplanting)

L – III = Location-III *viz*. Palampur (3^rd ^date of transplanting; 15 days after the normal transplanting)

L – IV = Location-IV *viz*. Rice Research Station, Malan (CSK HPAU)

L – V = Location-V *viz*. Farmers' fields, Pharer.

### Average comparison of prediction accuracy

Overall, the r^2 ^and percent mean absolute error showed better improvement with SVM based validation tests as compared to the REG, BPNN and GRNN approaches (Table 3). In case of 'cross-location' models, maximum coefficient of determination (r^2 ^= 0.33) was observed for the year 2000 as compared to the other years performance with the % MAE value of 67.01 with REG approach which improved to r^2 ^= 0.39 and % MAE = 51.68 when BPNN-based validation approach was followed. With GRNN, the r^2 ^showed an increase of about 8% in average r^2 ^value (0.47) with about 11% improvement in its respective average % MAE (40.20). The SVM-based validation approach again revealed an increase of 8% in average r^2 ^value to 0.55 and simultaneous improvement in its % MAE also by about 7% (33.74). Similar improvement in average r^2 ^and % MAE values was also observed in 'cross-year' models validation. The maximum average coefficient of determination (r^2 ^= 0.49) was observed for location-IV (Rice Research Station, Malan) showing 68.29% mean absolute error with REG-based validation approach which increased by 6% to r^2 ^= 0.55 and % MAE also improved by about 17% (50.85) with BPNN-based validation. The average r^2 ^further increased by 8% to 0.63 and the % MAE also improved by 9% to 41.44 with GRNN. However, the SVM-based validation again increased the average r^2 ^to 0.73, a jump of about 10% with simultaneous improvement by about 7% in its respective % MAE also (34.90).

**Table 3:** Overall comparison of multiple regression (REG), backpropagation neural network (BPNN), generalized regression neural network (GRNN) and support vector machine (SVM) based prediction accuracy of rice blast severity measured as average correlation coefficient (r), coefficient of determination (r^2^) and percent mean absolute error (%MAE) of observed value for 'cross-location' and 'cross-year' models.

**Model(s)**	**Multiple Regression (REG)**			**Artificial Neural Network (ANN)**						**Support Vector Machine (SVM)**		
	
				**BPNN**			**GRNN**					
	
	**r**	**r**^**2**^	**%MAE**	**r**	**r**^**2**^	**%MAE**	**r**	**r**^**2**^	**%MAE**	**r**	**r**^**2**^	**%MAE**
**Cross-location models**												
2000	0.57	0.33	67.01	0.61	0.39	51.68	0.67	0.47	40.20	0.73	0.55	33.74
2001	0.44	0.23	77.95	0.50	0.29	66.42	0.62	0.45	64.51	0.70	0.54	48.93
2002	0.50	0.29	97.48	0.64	0.45	94.04	0.66	0.49	83.87	0.69	0.58	57.09
2003	0.48	0.26	66.76	0.58	0.34	58.03	0.68	0.48	53.28	0.82	0.67	41.37
2004	0.43	0.22	78.51	0.47	0.28	60.38	0.65	0.44	49.43	0.78	0.62	39.44

**Average**	**0.48**	**0.27**	**77.54**	**0.56**	**0.35**	**66.11**	**0.66**	**0.47**	**58.26**	**0.74**	**0.59**	**44.12**

**Cross-year models**												
Location-I	0.42	0.21	65.96	0.51	0.29	61.12	0.60	0.37	55.93	0.69	0.48	47.97
Location-II	0.40	0.20	73.29	0.41	0.23	59.66	0.59	0.40	54.21	0.70	0.51	40.98
Location-III	0.42	0.22	56.11	0.62	0.39	38.41	0.76	0.59	33.10	0.78	0.62	25.85
Location-IV	0.68	0.49	68.29	0.73	0.55	50.85	0.78	0.63	41.44	0.85	0.73	34.90
Location-V	0.59	0.37	63.46	0.73	0.54	51.15	0.76	0.58	46.79	0.84	0.72	33.62

**Average**	**0.50**	**0.30**	**65.42**	**0.60**	**0.40**	**52.24**	**0.70**	**0.51**	**46.30**	**0.77**	**0.61**	**36.66**

where,

L – I = Location-I *viz*. Palampur (1^st ^date of transplanting; 15 days prior to normal transplanting)

L – II = Location-II *viz*. Palampur (2^nd ^date of transplanting; normal time of transplanting)

L – III = Location-III *viz*. Palampur (3^rd ^date of transplanting; 15 days after the normal transplanting)

L – IV = Location-IV *viz*. Rice Research Station, Malan (CSK HPAU)

L – V = Location-V *viz*. Farmers' fields, Pharer.

In order to have overall comparison of all the four approaches followed, the overall r^2 ^value averaged over all the years for 'cross-location' models with REG was observed to be 0.27 with its corresponding % MAE as 77.54. When BPNN was applied, the overall r^2 ^increased by 8% to 0.35 and % MAE (66.11) also improved by about 11%. The GRNN further increased the r^2 ^to 0.47, an increase of 12% with 8% improvement in its respective % MAE (58.26). However, the SVM gave the best reachable average r^2 ^value of 0.59, again an increase by 12% with its corresponding % MAE value of 44.12, which also improved drastically by about 14%. For 'cross-year' models, the average r^2 ^was found to be 0.30 with its respective % MAE of 65.42, which showed an increase of 10% (r^2 ^= 0.40) with BPNN with simultaneous 13% improvement in its % MAE (52.24) also. Further, the GRNN-based validation increased the average r^2 ^(0.51) by 11% and improved % MAE by about 6% (46.30). The SVM-based validation approach again proved its supremacy over the other three approaches by increasing the r^2 ^further to 0.61, an increase by 10%. The respective % MAE (36.66) also improved by about 10%. The 'cross-year' models showed slightly higher coefficient of determination and least percent mean absolute error values with all the four approaches followed in this study indicating that 'cross-year' predictions are better with respect to their accuracy and confidence level as compared to the 'cross-location' predictions.

### Best predictor variables

For 'cross-location' validation, the best SVM model was observed during 2001 with location-V as test data (farmers' fields, Pharer) with the highest correlation coefficient of 0.98 and percent mean absolute error of 66.40. The performance of this model was again tested by excluding each weather variable at a time by repeated training and testing. The results revealed that rainfall was most influential in predicting the disease followed by rainy days/week, minimum relative humidity, maximum relative humidity, minimum temperature and maximum temperature (Table 4). Similarly, for 'cross-year' models, the best SVM model was observed at location-V (farmers' fields, Pharer) with the year 2000 as test data with the highest correlation coefficient of 0.98 and least percent mean absolute error of 15.23. The rainfall was again found to be the best predictor variable. However, the second most influential variable was found to be minimum relative humidity followed by maximum relative humidity, minimum temperature, maximum temperature and rainy days/week. Overall, the rainfall was observed to be the best predictor among the weather variables followed by relative humidity and rainy days/week. The temperature was found to have least effect on disease development.

**Table 4:** Identification of most influential predictor variables for the best 'cross-location' and 'cross-year' SVM models.

**Weather variable(s) excluded**	**Best SVM 'cross-year' model Correlation coefficient (r)**	**Best SVM 'cross-location' model Correlation coefficient (r)**
None excluded. All 6 variables (T_max_, T_min_, RH_max_, RH_min_, Rainfall, RD/week) included	0.979	0.982
Rainfall	0.789	0.949
Rainy days/week	0.974	0.966
Relative Humidity (minimum)	0.866	0.982
Relative Humidity (maximum)	0.953	0.984
Temperature (minimum)	0.968	0.983
Temperature (maximum)	0.973	0.984

**Figure 1:**
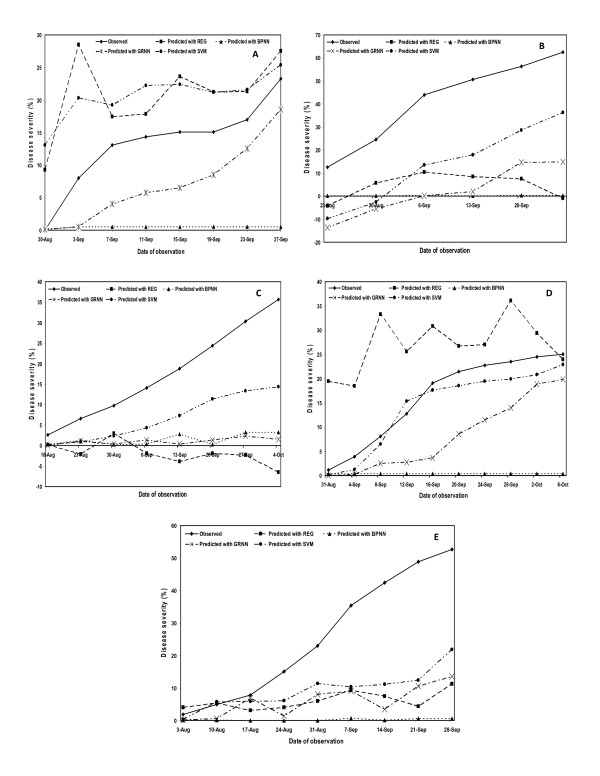
Observed and predicted mean disease severity based comparison of prediction accuracy of multiple regression (REG), backpropagation neural network (BPNN), generalized regression neural network (GRNN) and support vector machine (SVM) approaches for 'cross-location models' during year(s) 2000 (A); 2001 (B); 2002 (C); 2003 (D); and during 2004 (E).

**Figure 2:**
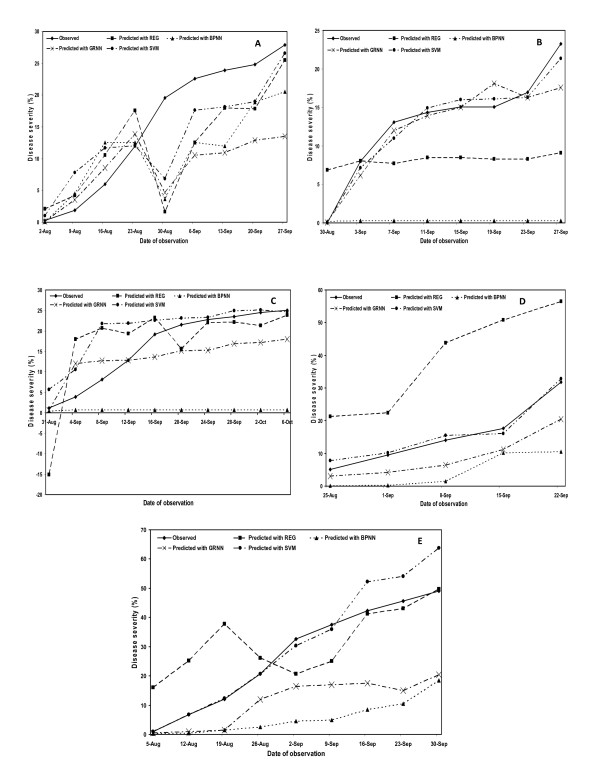
Observed and predicted mean disease severity based comparison of prediction accuracy of multiple regression (REG), backpropagation neural network (BPNN), generalized regression neural network (GRNN) and support vector machine (SVM) approaches for 'cross-year models' at Palampur-I (early transplanting) (A); at Palampur-II (normal transplanting) (B); at Palampur-III (late transplanting) (C); at Rice Research Station, Malan (D); and at farmers' fields, Pharer (E).

Finally, we selected the best prediction models for each of the four approaches followed *viz*. REG, BPNN, GRNN and SVM based on the maximum coefficient of determination and least percent mean absolute error values. Then, we plotted the observed and predicted mean disease severities to compare their prediction accuracy both for 'cross-year' as well as 'cross-location' models. Within the cross-year models, best prediction accuracy was observed for location-IV (Rice Research Station, Malan) and for location-V (farmers' fields, Pharer). Furthermore, best testing was observed with the year 2000 data at all the locations except for location-IV (RRS, Malan) where best validation was observed with 2001 data. In case of cross-location models, best prediction accuracy was observed for 2003 and 2004 years data (Figure 1, 2).

### Description of Web Server

**Figure 3:**
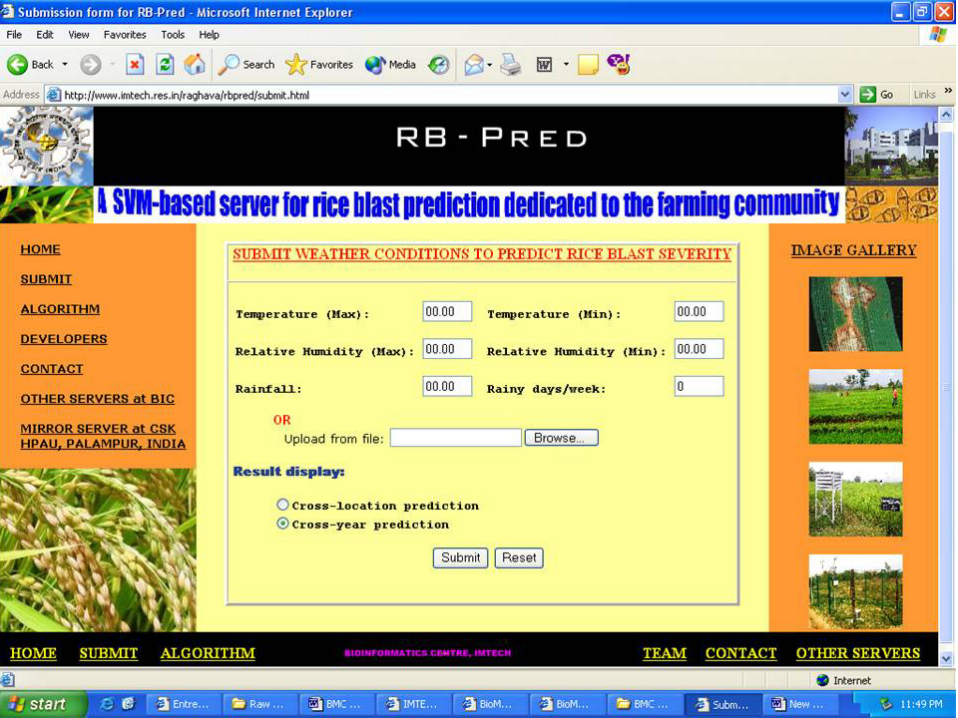
An overview of submission form for online prediction of rice blast severity with 'RB-Pred' web server.

A web-based server, RB-Pred, was developed to predict the severity percent of leaf blast. RB-Pred is beautifully designed and is a user-friendly and easy-to-use web server. Users just have to feed the recorded weather variables prevailing in their areas *viz*. temperature (maximum), temperature (minimum), relative humidity (maximum), relative humidity (minimum), rainfall and rainy days/week data in the 'submit' form of the server (Figure 3). Based on the maximum correlation coefficient and least percent mean absolute error, we have selected the two best models each for 'cross-location' as well as for 'cross-year' predictions. We have saved the model files of these best models in the server. When the user feed the weather variables, the server classifies them according to these model files and generates the predicted leaf blast severity (%) separately for cross-location as well as cross-year predictions. As the cross-year correlation was observed more than the cross-location validation, the predicted blast severity seems to be more accurate for 'cross-year' predictions as compared to the 'cross-location' predictions and thus, the default submit parameter was set on 'cross-year' models.

Although, the server is working quite well and will be beneficial to the plant pathologists and farmers; we are working towards the in-season real time weather based disease predictions where the web server will itself train the weather variables fed by the users and will ultimately, forecast the real time disease severity for their respective areas.

## Discussion

This work is the first initiative of applying support vector machine tools to developing weather-based prediction models for plant diseases as the usefulness of SVM over the existing neural networks (BPNN and GRNN) and the conventional multiple regression approaches in predicting plant diseases have not been reported yet from any part of the world. While previous research, particularly on rice blast prediction, has largely been focused on the conventional multiple regression approaches, no serious effort with new prediction softwares in improving the prediction accuracy of these models have been employed. There is an urgent need to manage rice blast by better understanding of the mathematical relationships between the environmental conditions and its specific stages of infection cycle. The rationale behind the use of early- and mid-season information to predict late-season rice blast severity lies in the fact that this disease is strongly influenced by environmental conditions favourable for initial inoculum production and initial infection. Thus, it was necessary to use other empirical approaches in an effort to understand the relationship between the environment and disease (rice blast) development. The results of the present study by using SVM-based regression approach have led to a better description of this relationship between the environmental conditions and disease level which could be useful for disease management.

Though, within the neural networks, the generalized regression neural network outperformed the backpropagation neural networks by about 20–30%. This is due to the fact that GRNN have greater understanding of model results (i.e. repeatable derivation of coefficients), less reliance on parameter optimization, and have minimum probability of being trapped in local minima during error gradient descent. Similar results of improvement in prediction accuracy through GRNN as compared to the BPNN have also been reported by Chtioui *et al*. for moisture prediction from simple micrometeorological data [27], Wolf and Francl [20,21] for prediction of tan spot and stagonospora blotch infection periods, and Francl [22] for modeling wetness duration. However, one major significant contribution of this study has been the improvement of r^2 ^and % MAE values with SVM as compared to the existing BPNN and GRNN and the conventional REG approaches. We demonstrated this by comparing all the four approaches in best possible combinations i.e. training and testing of both cross-location as well as cross-year prediction models. All the locations/years' data showed improved r^2 ^and least % MAE values with SVM based validation procedures indicating that SVM holds better supremacy over the other three modeling techniques. The advantages of using SVM over the other methods lies in the fact that SVMs are known to be robust when one has a sparsely filled high dimensional dataset as shown in some of the earlier studies [41-43]. It maps data into a high dimensional space via kernels where a linear decision boundary is constructed. Such boundary corresponds to a non-linear one in the input space [28,44]. Some other most remarkable advantages of SVMs are the absence of local minima, the sparseness of the solution, and the use of the kernel-induced feature spaces [42]. The flexibility allowed by this modeling approach makes it possible to fit non-linear relationships and complex interactions between variables without requiring complex transformations of variables and trial-and-error searches for interactions as required in neural networks. For a system like leaf blast of rice for which the relationships between the environment and disease development are not fully understood with conventional statistical techniques, SVM serves as an excellent tool for developing prediction models. Previous attempts to predict the severity of this disease using REG approaches yielded results inferior to those being reported in this study using SVM.

While previous research has largely used data from the same site [10,12,14,45], the work reported here demonstrated that data from some or all field sites could be used for cross-sites prediction with equal accuracy as that with the within-site prediction models. All the REG, BPNN, GRNN and SVM models were used to predict rice leaf blast severity for locations that were never used in model training/development. Training and testing sites were geographically distant and were spread over district Kangra of Himachal Pradesh (India). Both cross-year as well as cross-location wise training and testing of prediction models was done in order to compare their results for efficient validation across the two procedures. In general, the cross-year models showed slightly higher coefficient of determination and lower percent mean absolute error values as compared to the cross-location models during all the validation approaches followed in this study *viz*. REG, BPNN, GRNN and SVM. Moreover, when the observed and predicted disease severity values were plotted for both the models, the cross-year models showed less deviation between the observed and predicted curves as compared to the cross-location models (Figure 1, 2). This indicated that the 'cross-year' models (models validated across the years at same location) are better in terms of their prediction accuracy to the 'cross-location' models (models validated across the locations in the same year). In other words, we can say that location-specific models have better predictability and higher confidence of prediction.

Moreover, despite the rigorous and stringent validation procedures followed in this study for all the four approaches, the multi-location data showed more robustness and usefulness over a broad range of field sites. This important contribution also demonstrates the obvious underlying principle that the basic quantitative relationship between rice blast development and weather does not change from site to site, given that an inoculum source of a virulent pathogen and a susceptible host is present. However, using data only from locations with similar characteristics may be useful but these models essentially become location-specific and lose application over a broad range of locations. That's why the plotted observed and predicted disease severities showed more deviation in some cases for 'cross-location' models even with SVM as compared to the 'cross-year' models (Figure 1, 2). Moreover, these prediction techniques are based on various mathematical functions which try to predict the biological functions as accurately as they can, but it is difficult to predict the actual values with hundred per cent accuracy as the environmental factor, which is highly variable, always play a major role in disease predictions. However, our main concern in the present investigation was to evaluate the status of currently available approaches for predicting plant diseases in comparison to the latest prediction techniques that are still unexploited like SVM in this case. It was observed that SVM performed better than BPNN, GRNN and REG on the dataset used in this study. It does not indicate that in general, SVM is better than neural networks or the other methods. Our analysis on the present dataset revealed better prediction accuracy with SVM over all the years and locations as compared to the REG, BPNN and GRNN-based validation approaches which is an important and significant contribution of this study.

## Conclusion

The application of SVM models for plant disease prediction as revealed from the present case study on rice blast prediction makes them a useful tool for future forecasting models, and combining aspects of SVM and other established statistical tools may offer a more flexible option for the future. Experimental approaches, linked with dynamic models, are more adequate approaches allowing a better understanding of the dynamics of the systems. To date, only multiple regression and neural networks are being used extensively for forecasting plant diseases in various parts of the world. The higher predictive accuracy by latest machine learning techniques like SVM as demonstrated in the present study will generate more efficient prediction models which will help explain the factors that govern disease epidemics, and help in the design of control systems that minimize yield losses. Rice blast disease causes between 11% and 30% crop losses annually. This represents a loss of 157 million tonnes of rice [46]. Progression of the disease varies in different locations and years, depending mainly on weather conditions. Therefore, forecasting of blast epidemics is necessary if growers are to prevent severe yield losses caused by the disease [47]. Growers many want to know when the disease will start, how severe the epidemic will be, whether fungicides should be applied, and if so, when. For this, scientists are using computer modeling and simulation to synthesize and understand this complex pathosystem. However, there is no online disease prediction tool available that have potential to guide the farmers and researchers to develop various strategies for efficient control of plant diseases by timely forecasting of disease occurrence. 'RB-Pred' web-based server, which is freely available, is an initiative in this direction for forecasting leaf blast severity based on the weather variables which may help the farmers and plant pathologists in their decision making process. Based on the maximum r^2 ^and least % MAE values, the best prediction models developed with these four approaches followed in this study are being selected and a practical management program (web-based server) for rice blast is being developed at this centre using these models. Requiring in-season weather data as input, this server will directly serve to assess the risk of disease (rice blast) epidemics and recommending timely application of fungicides. Thus, the present web server could play an important tool in integrated rice blast management system for direct application to plant pathologists, academicians and farmers which will ultimately have direct economic impact in terms of (i) reduction in expenses to develop new fungicides, (ii) development of target-site specific and environment-friendly fungicides, and (iii) increase in savings by reducing fungicide application.

## Methods

### Data set

Data on weather and rice leaf blast severity were obtained from one year historical data for the year 2000 and from a NATP project entitled "Development of weather based forewarning systems for crop pests and diseases" which was implemented in the department of plant pathology, CSK Himachal Pradesh Agricultural University, Palampur from 2001 to 2004. During this period (one year historical and four years of NATP project), five different locations were selected *viz*. department of plant pathology experimental farm, CSK Himachal Pradesh Agricultural University, Palampur with three dates of sowing i.e. 15 days early to normal sowing (Location-I), normal sowing (Location-II), 15 days after the normal sowing (Location-III); Rice Research Station, Malan (Location-IV) and at farmers' fields, Pharer (Location-V), in order to generate epidemics of different severity and to represent as many of the various combinations of variables likely to influence the development of leaf blast as possible. The field experiments were conducted by sowing a blast-susceptible commercial variety, Himalaya 2216 at these five locations with a plot size of 300 m^2 ^for each sowing with a spacing of 15 × 15 cm. Transplanting of local varieties at location-V was done in the first fortnight of July. Observations were recorded in 25 points of 1 m^2 ^each marked at random. In each 1 m^2^, 10 randomly selected hills were tagged and observations on leaf blast were recorded at regular intervals as per the Standard Evaluation System of International Rice Research Institute (IRRI), Philippines.

Data on meteorological variables such as maximum and minimum temperature, maximum and minimum relative humidity, rainfall and rainy days per week were recorded daily with Automatic Micro Weather Station (UNIDATA, Australia) at Palampur and with thermo-hygrographs at Rice Research Station, Malan and farmers' fields at Pharer. Weekly averages of these weather variables were calculated for correlation studies, to determine their role in the development of rice blast epidemic.

### Model development and evaluation

#### (i) Multiple regression (REG)

The REG models were developed according to the general multiple regression equation:

Y^
 MathType@MTEF@5@5@+=feaafiart1ev1aaatCvAUfKttLearuWrP9MDH5MBPbIqV92AaeXatLxBI9gBaebbnrfifHhDYfgasaacH8akY=wiFfYdH8Gipec8Eeeu0xXdbba9frFj0=OqFfea0dXdd9vqai=hGuQ8kuc9pgc9s8qqaq=dirpe0xb9q8qiLsFr0=vr0=vr0dc8meaabaqaciaacaGaaeqabaqabeGadaaakeaacuqGzbqwgaqcaaaa@2DF5@ = a + b_1 _X_1 _+ b_2 _X_2 _+.........+ b_n _X_n_

where, a = intercept, b_n _= slope of line (the partial regression coefficient value), and X_n _= independent variable.

In this equation, the regression coefficients (or *B *coefficients) represent the *independent *contributions of each independent variable to the prediction of the dependent variable. Another way to express this fact is to say that, for example, variable *X*_1 _is correlated with the *Y *variable, after controlling for all other independent variables. SPSS software (SPSS Inc., Chicago, IL) was used to perform multiple regression analysis to develop the disease prediction models, where the leaf blast severity was used as the dependent variable and the weekly average of various weather variables 1 week prior to disease assessment *viz*. maximum temperature (X_1_), minimum temperature (X_2_), maximum relative humidity (X_3_), minimum relative humidity (X_4_), rainfall (X_5_) and rainy days per week (X_6_) were used as independent variables.

#### (ii) Artificial neural network (ANN)

ANN applications in the agricultural sciences have been researched for a wide range of classification, optimization and prediction problems. There are several types of ANN employing different types of architectures. The two broad architectures of ANN, feed-forward backpropagation neural network (BPNN) and the generalized regression neural network (GRNN) being widely used in regression based prediction problems are briefly discussed.

##### Feed-forward backpropagation neural network (BPNN)

As described by De Wolf and Francl [20,21] and Francl [22], a feed-forward BPNN generally consists of nodes or processing elements arranged in at least three layers (input, hidden and output). Within each layer, the nodes contain (user-defined) mathematical functions (activation functions) used to process the data before passing them on to the nodes in the adjacent layer. Each input variable is fed into the network via a separate node in the input layer. The nodes in the input layer are connected to nodes in hidden layer, and the nodes in the hidden layer are connected to nodes in the output layer by way of weights. Weights are analogous to coefficients in regression modeling. Information is passed (fed) through the network from the input layer to the output layer (forward) via the hidden layer and the connection weights, hence the term feed-forward. Following further processing in the output layer, network-estimated outputs are generated and compared with actual outputs, and errors are calculated (based on the difference between the actual and network outputs). The errors then are fed (propagated) backward (from output layer to input layer) through the network (error back-propagation), adjusting the connection weights so as to minimize the difference between the estimated and the actual outputs. The predictors and responses are presented to the network repeatedly (training) and, after each passage through the network, weights are adjusted. Through this iterative process, the network "learns" the relationship between the predictor and response variables and, when presented with a new set of inputs (validation), it is capable of predicting the outcome based on this relationship. BPNN models were developed by implementing Stuttgart Neural Network Simulator, SNNS version 4.2 [48].

##### Generalized regression neural network (GRNN)

General regression neural networks, a second type of neural network developed by Specht, do not require the optimization of multiple parameters that is required in BPNN [25]. It has a radial basis layer and a special linear layer which is similar to the radial basis network, but has a slightly different second layer. In GRNN, each set of input observations, *I*_1 _to *I*_i_, is associated one-to-one with an intermediate node, forming a pattern layer. The GRNN models estimate appropriate model coefficients with a single pass of the data used in model development which assign a non-parametric probability density function, in this case a Gaussian kernel, with width *s*, for each sample of independent and dependent variables. An appropriate value for *s *is determined empirically based on mean square error between observed and estimated dependent variables. A joint probability estimate is converted to a conditional mean of dependent variables given the sample of independent variables, and the predicted value of dependent variables for future independent variables is assigned the most probable value given data used to develop the model. The GRNN models were developed using the MATLAB (The MathWorks Inc., Natick, MA) software.

#### (iii) Support Vector Machine (SVM)

SVMs are universal approximators based on statistical learning and optimization theory which supports both regression and classification tasks and can handle multiple, continuous and categorical variables. To construct an optimal hyperplane, SVM employees an iterative training algorithm, which is used to minimize an error function. For the application of SVM, the complete theory can be found in Vapnik's monographs [29,30]. However, during this investigation, our main goal was to compare the performance of REG, BPNN and GRNN with SVM-based regression approaches to estimate the functional dependence of the dependent variable *Y *on a set of independent variables *X*. Therefore, we briefly discuss here the summary of SVM (regression).

##### Support Vector Regression (SVR)

SVM can be applied not only to classification problems but also to the case of regression [49]. Still it contains all the main features that characterize maximum margin algorithm: a non-linear function is leaned by linear learning machine mapping into high-dimensional kernel induced feature space [50]. In a regression SVM, we estimate the functional dependence of the dependent variable *Y *on a set of independent variables *X*. It assumes, like other regression problems, that the relationship between the independent and dependent variables is given by a deterministic function **f **plus the addition of some additive noise. The task is then to find a functional form for f that can correctly predict new cases that the SVM has not been presented with before. This can be achieved by training the SVM model on a sample set, i.e. training set, a process that involves, like classification and the sequential optimization of an error function. All other additional information regarding error function(s) and kernels used in SVM have been described in the supplementary material (see Additional file 1). In the present investigation, the public domain software, SVM_light was used, which implements SVM [51].

### Cross Validation

The standard test for measuring the predictive accuracy we used was a Cross Validation (CV) test for all the REG, BPNN, GRNN and SVM approaches. CV measures the performance of the prediction system in a self-consistent way by systematically leaving out a few datasets during the training process and testing the trained prediction system against those left-out datasets. Compared to the test on independent dataset, CV has less bias and better predictive and generalization power. The predictive ability of the models generated from all the approaches was tested by performing the cross validation test at all the five locations under study. Furthermore, the CV test was performed both between the locations as well as between the years, so as to check whether location-wise prediction is more accurate or the year-wise prediction is more efficient. Firstly, each location within a particular year was dropped in turn and the daily severity class of the dropped location was predicted using models developed from the remaining four locations. These were named as "cross-location models". Similarly, the analysis was performed for cross-year validation i.e. within a location, each year was dropped in rotation and the daily severity class of leaf blast of the dropped year was predicted using models developed from the remaining four years data. These were termed as "cross-year models". We briefly discuss here the validation procedures followed for the REG, BPNN, GRNN and SVM-based models separately.

#### (i) Multiple regression models validation

Each case was defined as an observation with a unique combination of year, location and values for predictor variables. For cross-location models, a random sample by combining data of four locations was used for performing the multiple regression analysis and the data for remaining fifth location was used for testing (validation). This was done five times by dropping each location at a time. Similar procedure was followed for cross-year models validation by dropping each year data at a time and performing multiple regression on combined sample of other four years data. Thus, for validation, the independent weather variables *viz*. maximum temperature (X_1_), minimum temperature (X_2_), maximum relative humidity (X_3_), minimum relative humidity (X_4_), rainfall (X_5_) and rainy days per week (X_6_) of the remaining test dataset were fed into the above REG equation and the predicted disease severity values (Y^
 MathType@MTEF@5@5@+=feaafiart1ev1aaatCvAUfKttLearuWrP9MDH5MBPbIqV92AaeXatLxBI9gBaebbnrfifHhDYfgasaacH8akY=wiFfYdH8Gipec8Eeeu0xXdbba9frFj0=OqFfea0dXdd9vqai=hGuQ8kuc9pgc9s8qqaq=dirpe0xb9q8qiLsFr0=vr0=vr0dc8meaabaqaciaacaGaaeqabaqabeGadaaakeaacuqGzbqwgaqcaaaa@2DF5@) were calculated at 3-day interval for the whole crop season at Palampur (location – I, II, III) and at weekly intervals for Rice Research Station, Malan (location – IV) and farmers' fields, Pharer (location – V). Then, we calculated the correlation coefficient (r) between the observed and predicted disease severity values. Actual prediction accuracy of these REG models was thus, determined on the basis of coefficient of determination (r^2^) and percent mean absolute error (% MAE) of the actual values. The additional details regarding formulae adopted for calculating r, r^2 ^and % MAE values can be found in the supplementary material (see Additional file 1).

#### (ii) BPNN, GRNN and SVM models validation

For all these three approaches, similar procedure was followed for sampling the data according to cross-validation approach as done for REG-based validation. A random sample of combined four years/locations was used to train the network and the remaining fifth year/location data for testing (validation). In this way, the predictors and responses were presented to the network repeatedly (training) and, after each passage through the network, weights were adjusted. Through this iterative process, the network "learns" the relationship between the predictor and response variables and, when presented with a new set of inputs (validation), it is capable of predicting the outcome based on this relationship. The iterations were continued for generating prediction models until the best model was found on the basis of maximum correlation coefficient between the observed and predicted disease severities. Training was stopped and the network was saved on the best test set; that is, when the error (difference between actual and predicted severity) for the test set was minimized. Separate sets of models were developed for each set of input variables at all the five locations both within cross-location and cross-year models. These validation cases were then used to assess the overall performance of the models by using the correlation coefficient values between the predicted and observed disease severity which were analyzed for determining the r^2 ^and % MAE of the actual value as the final measures of prediction accuracy.

The prediction models which showed highest r^2 ^with the least % MAE were adjudged the best prediction models for each of the four approaches followed. All other additional information on data preprocessing such as scaling or normalization, learning rules, weights adjustment etc. can be found in the supplementary material (see Additional file 1).

### Identification of best predictor variables

Based on the maximum correlation coefficient and least percent mean absolute error values, the best SVM-based models were selected for 'cross-location' as well as 'cross-year' models. The performance of these models was again validated through training and testing by excluding one weather variable at a time in order to identify the most influential weather variable(s) in the SVM model. During validation, where least correlation coefficient was found, the corresponding excluded variable was declared as the most influential predictor variable in the model. Similarly, by excluding each variable at a time, the decrease in correlation coefficient was judged and the series of best predictors was identified in decreasing order accordingly for both the 'cross-location' and 'cross-year' models.

## List of abbreviations used

REG, Multiple regression; ANN, Artificial neural network; BPNN, Back-propagation neural network; GRNN, Generalized regression neural network; SVM, Support vector machine; SVR, Support vector regression; RBF, Radial basis function; CV, Cross validation; %MAE, Percent mean absolute error; NATP, National Agricultural Technology Project.

## Authors' contributions

RK carried out the data analysis and interpretation, developed computer programs, wrote the manuscript and developed the web server. ASK provided the five years data and revised the manuscript critically for important intellectual as well as professional content. GPSR conceived and coordinated the project, guided its conception and design, helped in interpretation of data, refined the drafted manuscript and gave overall supervision to the project. All authors read and approved the final manuscript.

## Supplementary Material

Additional file 1 Supplementary material. All the additional information regarding evaluation of methods, data preprocessing and normalization, steps followed for various approaches etc. have been provided in this additional file. Click here for file
