# Supplementary material for: Machine learning techniques in disease forecasting: a case study on rice blast prediction
Source: BMC Bioinformatics. 2006 Nov 3;7:485. doi: 10.1186/1471-2105-7-485 (PMC1647291; doi:10.1186/1471-2105-7-485)
Supplement: Additional file 1 — Supplementary material. All the additional information regarding evaluation of methods, data preprocessing and normalization, steps followed for various approaches etc. have been provided in this additional file. [file 1471-2105-7-485-S1.pdf]

Supplementary Material

Kaundal, R., Kapoor, A.S. and Raghava, G.P.S. (2006) Machine learning techniques in disease forecasting: a case study on rice blast prediction. *BMC Bioinformatics* (accepted)

1. Evaluation of Methods

The performances of various approaches followed in this study *viz*. REG, BPNN, GRNN and SVM were evaluated using following three mathematical parameters:

Correlation coefficient: The quantity ***r***, called the *linear correlation coefficient*, measures the strength and the direction of a linear relationship between two variables. The linear correlation coefficient is sometimes referred to as the *Pearson product moment correlation coefficient* in honour of its developer Karl Pearson. The mathematical formula used for computing ***r*** is:

$$r = \frac{\Sigma XY - \frac{\Sigma X \Sigma Y}{N}}{\sqrt{\left(\Sigma X^2 - \frac{(\Sigma X)^2}{N}\right)\left(\Sigma Y^2 - \frac{(\Sigma Y)^2}{N}\right)}}$$

where, *X* and *Y* are observed and predicted value of disease severity, respectively. N is the total number of observations in the data set at a particular location / year. The value of ***r*** is such that $-1 \leq r \leq +1$. The + and – signs are used for positive linear correlations and negative linear correlations, respectively.

Coefficient of determination: The *coefficient of determination,* r^2, is simply the square of correlation coefficient but it is very useful because it gives the proportion of the variance (fluctuation) of one variable that is predictable from the other variable. It is a measure that allows us to determine how certain one can be in making predictions from a certain model. Thus, the r^2 represents the ratio of the explained variation to the total variation. The *coefficient of determination* is such that $0 \leq r^2 \leq 1$, and denotes the strength of the linear association between *X* and *Y*. It represents the percent of data that is the closest to the line of best fit. For example, if r = 0.856, then r^2 = 0.733, which means that 73% of the total variation in *Y* can be explained by the linear relationship between *X* and *Y*. The other 27% of the total variation in *Y* remains unexplained. Thus, the r^2 is a measure of how well the regression line represents the data. If the regression

line passes exactly through every point on the scatter plot, it would be able to explain all of the variation. The farther the line is from the points, the less it is able to explain.

Percent mean absolute error: Though correlation coefficient and determination of coefficient tell the relationship between two variables, one can not find out if the magnitude of two values is same or not. In order to calculate the error between predicted and observed value, we computed percent mean absolute error (MAE) of the observed value, which provides chances of error in prediction. Following equation was used for calculating MAE:

$$MAE = \frac{MeanError}{MeanObservedError} = \frac{\frac{\sum_{i=1}^{N}(Y_i - X_i)}{N}}{\frac{\sum_{i=1}^{N} X_i}{N}} = \frac{\sum_{i=1}^{N}(Y_i - X_i)}{\sum_{i=1}^{N} X_i}$$

where X_i and Y_i are observed and predicted disease severity for i^{th} observation; N is the total number of observations at a particular location / year. The percent mean absolute error (% MAE) was computed by multiplying MAE with 100.

2. Support Vector Machine

In this study, the regression module of support vector machine (SVM) has been introduced to predict severity of rice blast disease. The basic features of SVM and support vector regression (SVR) are briefly discussed in the main article. However, there are certain error functions and kernels which are to be optimized during the SVR process. These error functions and kernels are briefly discussed here.

Depending on the definition of this error function, two types of SVM models can be recognized:

Regression SVM Type 1: For this type of SVM the error function is:

$$\frac{1}{2}w^T w + C\sum_{i=1}^{N}\xi_i + C\sum_{i=1}^{N}\xi^*{}_i$$

which we minimize subject to:

$$w^T\phi(x_i) + b - y_i \leq \varepsilon + \xi_i^*$$
$$y_i - w^T\phi(x_i) - b_i \leq \varepsilon + \xi_i$$
$$\xi_i, \xi_i^* \geq 0, i = 1, \ldots, N$$

Regression SVM Type 2

For this SVM model, the error function is given by:

$$\frac{1}{2}w^T w - C\left(v\varepsilon + \frac{1}{N}\sum_{i=1}^{N}\left(\xi_i + \xi_i^*\right)\right)$$

which we minimize subject to:

$$\left(w^T\phi(x_i)+b\right)-y_i \leq \varepsilon + \xi_i$$
$$y_i - \left(w^T\phi(x_i)+b_i\right) \leq \varepsilon + \xi^*{}_i$$
$$\xi_i, \xi^*{}_i \geq 0, i = 1,\ldots,N, \varepsilon \geq 0$$

In this study, we tried to find a regression formula based on training samples presented by M observations. The input $\mathbf{x}$ is first mapped onto a m-dimensional feature space using some fixed (nonlinear) mapping, and then a linear model is constructed in this feature space. Using mathematical notation [30, 49], the linear model (in the feature space) $f(\mathbf{x},\omega)$ is given by

$$f(\mathbf{x},\omega) = \sum_{j=1}^{m}\omega_j g_j(\mathbf{x}) + b \qquad \text{(Equation – I)}$$

where $g_j(\mathbf{x}), j = 1,..., m$ denotes a set of nonlinear transformations, and b is the “bias” term. Often the data are assumed to be zero mean (this can be achieved by preprocessing), so the bias term is dropped.

The quality of estimation is measured by the loss function $L(y, f(\mathbf{x},\omega))$. SVM regression uses a new type of loss function called ε-insensitive loss function proposed by Vapnik [30]:

$$L_\varepsilon(y, f(\mathbf{x},\omega)) = \begin{cases} 0 & if\ |y - f(\mathbf{x},\omega)| \leq \varepsilon \\ |y - f(\mathbf{x},\omega)| - \varepsilon & otherwise \end{cases}$$

The empirical risk is:

$$R_{emp}(\omega) = \frac{1}{n}\sum_{i=1}^{n} L_\varepsilon(y_i, f(\mathbf{x}_i,\omega))$$

SVM regression performs linear regression in the high-dimension feature space using ε-insensitive loss and, at the same time, tries to reduce model complexity by minimizing $\|\omega\|^2$. This can be described by introducing (non-negative) slack variables ξ_i, ξ_i^* $i = 1,...n$, to measure the deviation of training samples

outside ε-insensitive zone. Thus, SVM regression is formulated as minimization of the following functional:

$$\min \quad \frac{1}{2} \| \omega \|^2 + C \sum_{i=1}^{n} (\xi_i + \xi_i^*)$$

$$\text{s.t.} \quad \begin{cases} y_i - f(\mathbf{x}_i, \omega) \le \varepsilon + \xi_i^* \\ f(\mathbf{x}_i, \omega) - y_i \le \varepsilon + \xi_i \\ \xi_i, \xi_i^* \ge 0, i = 1, ..., n \end{cases}$$

This optimization problem can be transformed into the dual problem and its solution is determined by rewriting the Equation-I as

$$f(\mathbf{x}) = \sum_{i=1}^{n_{SV}} (\alpha_i - \alpha_i^*) K(\mathbf{x}_i, \mathbf{x}) \quad \text{s.t. } 0 \le \alpha_i^* \le C, 0 \le \alpha_i \le C, \qquad \text{(Equation – II)}$$

where n_{SV} is the number of Support Vectors (SVs) and the kernel function

$$K(\mathbf{x}, \mathbf{x}_i) = \sum_{j=1}^{m} g_j(\mathbf{x}) g_j(\mathbf{x}_i)$$

Kernel Functions

It is well known that SVM generalization performance (estimation accuracy) depends on a good setting of C, ε and the kernel parameters. The problem of optimal parameter selection is further complicated by the fact that SVM model complexity (and hence its generalization performance) depends on all three parameters. There are a number of kernels that can be used in SVM models [50]. These include linear, polynomial, radial basis function (RBF) and sigmoid:

$$\phi = \begin{cases} x_i * x_j & \text{Linear} \\ (\gamma x_i x_j + \text{coefficient})^{\text{degree}} & \text{Polynomial} \\ \exp(-\gamma |x_i - x_j|^2) & \text{RBF} \\ \tanh(\gamma x_i x_j + \text{coefficient}) & \text{Sigmoid} \end{cases}$$

The RBF is by far the most popular choice of kernel types used in SVMs. This is mainly because of their localized and finite responses across the entire range of the real x- axis. One of the advantages of SVM, and SVR as the part of it, is that it can be used to avoid difficulties of using linear functions in the high dimensional feature space and optimization problem is transformed into dual convex quadratic programmes. In regression case, the loss function is used to penalize errors that usually lead to the sparse representation of the decision rule, giving significant algorithmic and representational advantages.

Once the support vectors, their Lagrange multipliers, and the bias b are determined from the training samples, Equation – II as described above can be used to predict the disease severities.

3. Artificial Neural Networks

Data preprocessing: In the present study, we used two widely used regression algorithms of ANN *viz*. back propagation neural network (BPNN) and generalized regression neural network (GRNN). Both are widely used as universal approximators. The network geometry determines the number of connection weights and how these are arranged. This is generally done by fixing the number of 'hidden layers' and choosing the number of 'nodes' in each of these layers. But before that, data preprocessing is an important step in developing a neural network application, which could affect model accuracy and results. The preprocessing/normalization helps in avoiding the dimensional dissimilarities of different input parameters. The variables have to be scaled in such a way as to be commensurate with the limits of the transfer function used in the output layer. In the present study, data used for both the BPNN and GRNN approaches was scaled in the range (0 – 1) by dividing all the observed disease severity values by the maximum disease severity in that particular location/year. This was done separately for each location and for each year data. In normalizing data, the goal is to ensure that the statistical distribution of values for each net input and output is roughly uniform. In addition, the values should be scaled to match the range of the input neurons. This means that along with any other transformations performed on network inputs, each input should be normalized as well.

Steps in BPNN: Backpropagation is a supervised learning technique used for training artificial neural networks. It is most useful for feed-forward networks (networks that have no feedback, or simply, that have no connections that loop). The term is an abbreviation for "backwards propagation of errors". Backpropagation requires that the transfer function used by the artificial neurons (or "nodes") be differentiable.

The BPNN algorithm in the present study was implemented through SNNS software. There are several steps in backpropagation training which are summarized as follows:

1. At first, create a network as

   name of file = filename.net

name of net = filename

size of window = 1

hidden nodes = optimize at various levels 5, 10, 15, 20…….

number of inputs = 6 (as there were six predictor weather variables in our study)

number of outputs = 1

2. Present a training sample to the neural network.
3. Compare the network's output to the desired output from that sample. Calculate the error in each output neuron.
4. For each neuron, calculate what the output should have been, and a *scaling factor*, how much lower or higher the output must be adjusted to match the desired output. This is the local error.
5. Adjust the weights of each neuron to lower the local error.
6. Assign "blame" for the local error to neurons at the previous level, giving greater responsibility to neurons connected by stronger weights.
7. Repeat the steps above on the neurons at the previous level, using each one's "blame" as its error.

As the algorithm's name implies, the errors (and therefore the learning) propagate backwards from the output nodes to the inner nodes. Backpropagation usually allows quick convergence on satisfactory local minima for error in the kind of networks to which it is suited. In addition, these computer neural network models are mainly described in terms of their architecture (patterns of connection) and in terms of the way they are trained (rules for modifying weights). So, in order to have efficient training for correct predictions, there are some set learning rules and momentums which are to be optimized regularly during the training process.

Learning rules

A simple linear network, with its fixed weights, is limited in the range of output vectors it can associate with input vectors. Hence, in addition to the network topology, an important component of most neural networks is a 'learning rule'. A learning rule allows the network to adjust its connection weights in order to

associate given input vectors with corresponding output vectors. During training periods, the 'input' vectors are repeatedly presented, and the 'weights' are adjusted according to the learning function (0.1, 0.01, 0.001…..), until the network learns the desired associations, i.e., until Y = weight * X. It is this ability to learn that is one of the most attractive features of neural networks.

Momentums

In addition to learning rules for training, there is another algorithm for BPNN networks that often provides faster convergence called steepest descent with momentum. 'Momentum' allows a network to respond not only to the local gradient, but also to recent trends in the error surface. Acting like a lowpass filter, momentum allows the network to ignore small features in the error surface. Without momentum, a network can get stuck in a shallow local minimum. With momentum, a network can slide through such a minimum. In the present study, we added momentum to backpropagation learning by making weight changes equal to the sum of a fraction of the last weight change and the new change suggested by the backpropagation rule. The magnitude of the effect that the last weight change is allowed to have is mediated by a momentum constant, which can be any number between 0 and 1. When the momentum constant is 0, a weight change is based solely on the gradient. When the momentum constant is 1, the new weight change is set to equal the last weight change and the gradient is simply ignored. The gradient is computed by summing the gradients calculated at each training example, and the weights and biases are only updated after all training examples have been presented.

Steps in GRNN: GRNN or Generalized regression neural network is a kind of normalized RBF network in which there is a hidden unit centered at every training case. These RBF units are called 'kernels' and are usually probability density functions such as the Gaussian. The hidden-to-output weights are just the target values, so the output is simply a weighted average of the target values of training cases close to the given input case. The only weights that need to be learned are the widths of the RBF units. These widths (often a single width is used) are called "smoothing parameters" or "bandwidths" and are usually chosen by cross-validation. In the present investigation, we used MATLAB software for GRNN-based predictions. The steps to be followed in MATLAB are briefly summarized as:

Design a generalized regression neural network

Syntax

```
net = newgrnn (P, T, spread)
```

Description

Generalized regression neural networks are a kind of radial basis network that is often used for function approximation. GRNNs can be designed very quickly.

`newgrnn(P,T,spread)` takes three inputs,

`P - R x Q` matrix of `Q` input vectors.

`T - S x Q` matrix of `Q` target class vectors.

`spread` - Spread of radial basis functions (distance an input vector must be from a neuron's weight vector), default = 1.0.

and this returns a new generalized regression neural network.

The larger the `spread`, the smoother the function approximation will be. To fit data very closely, use a `spread` smaller than the typical distance between input vectors. To fit the data more smoothly, use a larger `spread`.

Properties

`newgrnn` creates a two layer network. The first layer has many neurons as there are input/target vectors, calculates weighted inputs with `dist` and net input with `netprod`. The first layer operates just like the radial basis layer. Each neuron's weighted input is the distance between the input vector and its weight vector, calculated with `dist`. Each neuron's net input is the product of its weighted input with its bias, calculated with `netprod`. Each neuron's output is its net input passed through `radbas`.

If a neuron's weight vector is equal to the input vector (transposed), its weighted input will be 0, its net input will be 0, and its output will be 1. If a neuron's weight vector is a distance of spread from the input vector, its weighted input will be `spread`, and its net input will be sqrt (-log (0.5)) (or 0.8326). Therefore, its output will be 0.5.

The second layer has also many neurons as the input/target vectors calculated as `purelin` neurons, calculates weighted input with `normprod` and net inputs with `netsum`. Only the first layer has biases. `newgrnn` sets the first layer weights, and the first layer biases are all set to 0.8326/`spread`, resulting in radial basis functions that cross 0.5 at weighted inputs of +/- `spread`. Suppose we have an input vector **P** close to $\mathbf{P_i}$, one of the input vectors among the input vector/target pairs used in designing layer one weights. This input **P** produces a layer **1** $\mathbf{a^i}$ output close to 1. This leads to a layer 2 output close to $\mathbf{t_i}$, one of the targets used forming layer 2 weights.
